# Modified FlowCAM procedure for quantifying size distribution of zooplankton with sample recycling capacity

**DOI:** 10.1371/journal.pone.0175235

**Published:** 2017-04-06

**Authors:** Esther Wong, Akash R. Sastri, Fan-Sian Lin, Chih-hao Hsieh

**Affiliations:** 1 Institute of Oceanography, National Taiwan University, Taipei, Taiwan; 2 Division of Life Science, The Hong Kong University of Science and Technology, Clear Water Bay, Hong Kong; 3 Ocean Networks Canada and Biology Department, University of Victoria, Victoria, BC, Canada; 4 Institute of Ecology and Evolutionary Biology, Department of Life Science, National Taiwan University, Taipei, Taiwan; 5 Research Center for Environmental Changes, Academia Sinica, Taipei, Taiwan; 6 National Center for Theoretical Sciences, Taipei, Taiwan; CNRS, FRANCE

## Abstract

We have developed a modified FlowCAM procedure for efficiently quantifying the size distribution of zooplankton. The modified method offers the following new features: 1) prevents animals from settling and clogging with constant bubbling in the sample container; 2) prevents damage to sample animals and facilitates recycling by replacing the built-in peristaltic pump with an external syringe pump, in order to generate negative pressure, creates a steady flow by drawing air from the receiving conical flask (i.e. vacuum pump), and transfers plankton from the sample container toward the main flowcell of the imaging system and finally into the receiving flask; 3) aligns samples in advance of imaging and prevents clogging with an additional flowcell placed ahead of the main flowcell. These modifications were designed to overcome the difficulties applying the standard FlowCAM procedure to studies where the number of individuals per sample is small, and since the FlowCAM can only image a subset of a sample. Our effective recycling procedure allows users to pass the same sample through the FlowCAM many times (i.e. bootstrapping the sample) in order to generate a good size distribution. Although more advanced FlowCAM models are equipped with syringe pump and Field of View (FOV) flowcells which can image all particles passing through the flow field; we note that these advanced setups are very expensive, offer limited syringe and flowcell sizes, and do not guarantee recycling. In contrast, our modifications are inexpensive and flexible. Finally, we compared the biovolumes estimated by automated FlowCAM image analysis versus conventional manual measurements, and found that the size of an individual zooplankter can be estimated by the FlowCAM image system after ground truthing.

## Introduction

Body size has been recognized as the most critical trait determining metabolic rates of organisms; as a consequence, population traits, such as abundance, production, and turnover rate, all scale with size [1]. In addition, body size has been demonstrated to play an important role in mediating predator-prey interactions [1–3]. In fact, size has been designated as the “meta-trait” integrating several plankton functional traits into a single measurement [4, 5]. Since the pioneering works of Sheldon and colleagues [6, 7], size distribution has also figured prominently in the studies of plankton community structure and dynamics [3, 8–10].

There is active interest in developing efficient methods for obtaining size distributions of plankton [11–13], especially given the excellent potential for size structure to serve as a bio-indicator of environmental change. The relatively recent developments of automatic counting and measuring instruments bear several advantages over more conventional manual methods (i.e. microscopy and simple imaging software) for estimating plankton biovolume. Microscopic analysis tends to be time-consuming, highly repetitive, and often involves searching and measurement processes which can be subjective relative to the analyst. To reduce sample processing time and produce higher quality, consistent data, automated optical instruments integrated with imaging software have been developed to rapidly count and size plankton.

At present, the two most frequently used systems for automated plankton analysis are the ZooScan and FlowCAM. The ZooScan is suitable for imaging particles ranging in size from 200 μm to several centimetres [14] and the FlowCAM is preferable for analysing smaller plankton. In practice, scientists have used the FlowCAM to obtain cell counts [15, 16], size structure [3, 9, 13, 17], and community composition [13, 18]. Nonetheless, most of the researches using the FlowCAM have been limited to unicellular plankton, namely phytoplankton and protists (even though the FlowCAM manufacturer has suggested its application for measuring zooplankton for some time [18]). Recently, however, Le Bourg and colleagues [18] successfully estimated the abundance of small metazooplankton communities (80–1000 μm) using the FlowCAM and found results similar to those using a stereomicroscope.

While recognizing the utility of the FlowCAM to count and size mesozooplankton, several challenges remain. First, the FlowCAM only takes images of a subset of a sample. Second, processed samples are destroyed by the built-in peristaltic pump using the standard FlowCAM procedure. Third, the accuracy of zooplankton biovolume estimation based on the FlowCAM image analysis has not been evaluated. These issues are especially problematic if only a limited number of plankton specimens are available and so almost every particle needs to be measured, or if the samples are precious and need to be conserved. A practical case example is the need to estimate the growth rate of zooplankton using the artificial cohort method, in which the size distribution of zooplankton before and after incubation needs to be quantified [19]. In typical artificial cohort experiments, the number of individuals in each incubation container is limited [20]. Therefore, modifications to the FlowCAM settings and procedures are needed in order to recycle samples for studies in which plankton samples are limited. The development of a sample recycling capacity would allow repeated FlowCAM processing of the same sample in order to obtain a statistically robust size distribution in a non-destructive manner. We appreciate that the FlowCAM can be equipped with Field of View (FOV) flowcells which can image all particles passing the flow field; however, these FOV flowcells are very expensive, are of a limited variety of flowcell sizes, and do not guarantee sample recycling.

In this study, we developed a modified FlowCAM setup and procedure to achieve a non-destructive recycling capacity. Our modification is low cost and is applicable to any existing FlowCAM model and flowcell size. We also test the reliability of zooplankton biovolume estimation by automated FlowCAM image analysis. Here, we demonstrate the efficacy of our modified FlowCAM procedure using samples from artificial cohort incubations of copepods in the East China Sea.

## Modified FlowCAM procedure

**Fig 1:**
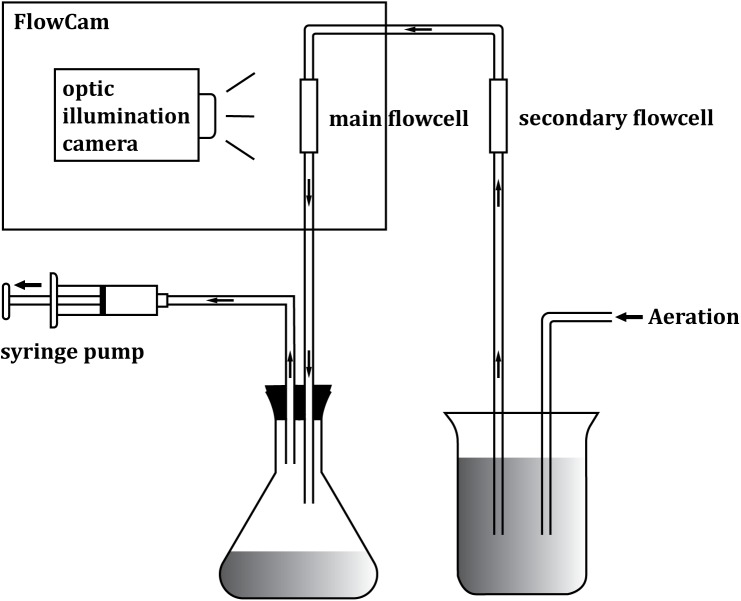
Schematic illustration of the modified FlowCAM procedure for optimizing FlowCAM capacity for zooplankton analysis. Modifications were made upon the standard FlowCAM setup: constant air bubbling (to prevent particles from settling and aggregating), a secondary flowcell (to force particles to align and to provide a window to monitor the occurrence of clogging), an external syringe pump, and a receiving conical flask (to archive sample recycling).

As a novel method for measuring copepod biovolume, we designed a new FlowCAM operational setup and procedure (Fig 1). Before carrying out the FlowCAM measurements, we removed formalin from the sample by gently washing the sample using distilled water 2 to 5 times to avoid image overlap due to agglomeration. Instead of loading the zooplankton sample directly into the FlowCAM, we first placed the zooplankton sample in a 250 ml glass beaker of distilled water. Air was then pumped into this diluted sample in order to generate a constant circulation of fluid and particles; as such, we prevented animals from settling and aggregating. We then used an external syringe pump, instead of the built-in peristaltic pump, to generate a negative pressure by drawing air from the receiving conical flask (Fig 1) in a manner analogous to a vacuum pump. The pump creates a steady flow, which directs plankton from the sample container toward the main flowcell of the imaging system. Note, we attached an additional (secondary) flowcell of the same model before the main flowcell; by doing so, particles were forced to align along the flow stream in the system. This secondary flowcell was also used to monitor for the occurrence of clogging during the experiments. Particles passing through the main flowcell were then imaged (as per the conventional FlowCAM setup). Finally, specimens were collected in the receiving conical flask for recycling. When necessary, this same procedure can be repeated several times on the same sample; this is analogous to bootstrapping the sample.

Note, a fixed, or universal, flow rate is not recommended; rather, the optimal flow rate should depend on the actual size range and shapes of the animals in the particular sample. We recommend adjusting the flow rate to a speed at which complete body images of individual copepods can be captured. To achieve this optimal speed, we suggest starting the FlowCAM analysis with a slow flow rate and then gradually increasing the rate to the point at which complete copepod body images can be captured without severe image duplication.

## Empirical demonstration of modified FlowCAM procedure

### Sample collection and artificial cohort experiments

**Fig 2:**
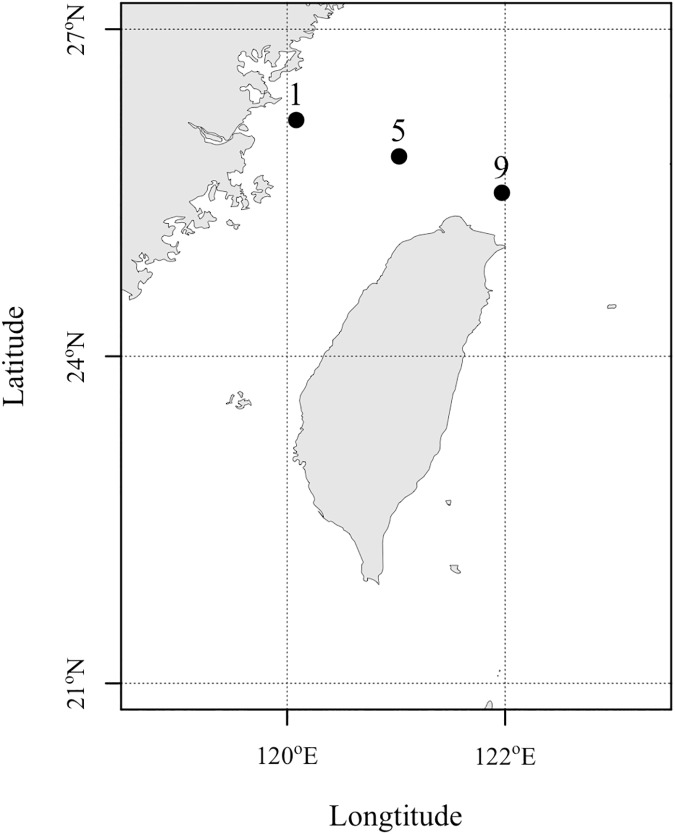
Map showing the three sampling stations in the East China Sea. Copepod nauplii and copepodites samples were respectively collected with 50 and 100 μm zooplankton net at 10 m depth at station 1, 5 and 9 in May, 2013.

We demonstrated the modified FlowCAM procedure using samples from artificial cohort incubation experiments of copepods in the East China Sea. Sampling was carried out on board the R/V Ocean Researcher II at three stations in the East China Sea (Fig 2) from May 5^th^ to 7^th^, 2013. The sampling and experiments of animals in this study requires no permit. For each sampling station, two separate Norpac zooplankton nets (with a ring diameter of 45 cm) with mesh size 50 and 100 μm were deployed to collect copepod nauplii and copepodites, respectively. The nets were set to 10 m depth and allowed to drift with the ship for 10 minutes. The contents of each net were then used for three replicate incubations, following the standard protocol of the artificial cohort method [19]. The samples before and after incubation were collected and then preserved in 5% formalin.

### FlowCAM settings

The size distribution of copepods in each sample was measured using the modified FlowCAM procedure, as described above. In this case study, images of copepods were captured using the FlowCAM autoimage mode, with an imaging rate of 20 frames per second. We used the flowcell with a 300 μm chamber depth and the 40x magnification microscope lens, for an optimum, factory defined particle size range of 30–300 μm. The flow rate was controlled by the pulling rate of the syringe pump, which was adjusted to an optimal speed such that complete body images of whole individual copepods were captured. As a reference, the size of the syringe used in this study is 100 ml with diameter 3.4 cm, and the general withdrawal rate of the syringe is 1.5–1.8 ml/min by the syringe pump.

### Evaluation of individual biovolume estimated by FlowCAM image analysis

**Fig 3:**
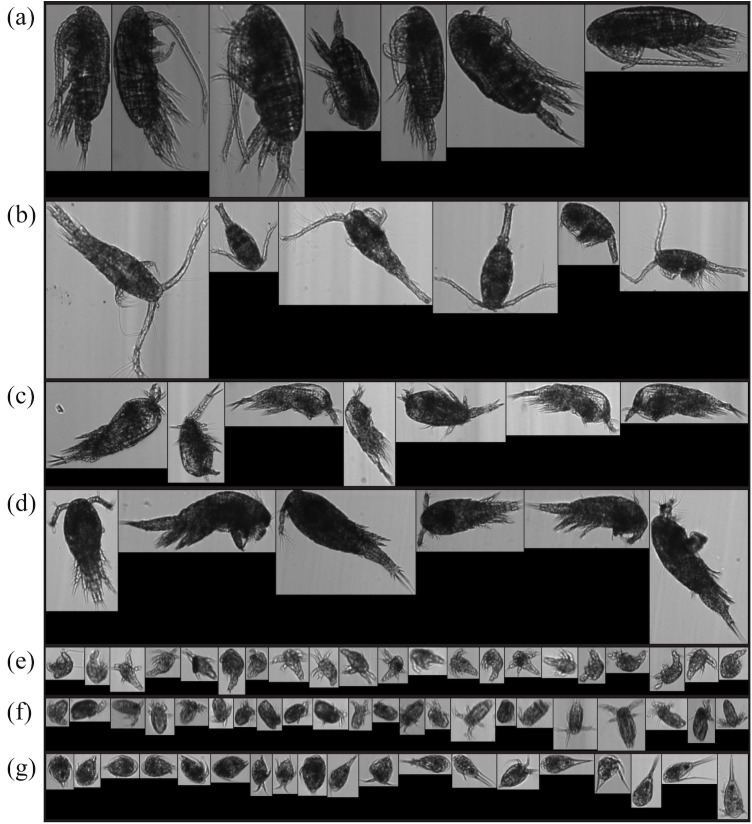
Examples of FlowCAM images of the 7 dominant copepod morphotypes. Examples from FlowCAM image libraries that were built prior to the semi-automatic classification for the 7 dominant copepod morphotypes: (a) calanoid copepodite, (b) oithonid (cyclopoid) copepodite, (c) corycaeid (poecilostomatoid) copepodite, (d) oncaeid (poecilostomatoid) copepodite, (e) calanoid nauplius, (f) cyclopoid nauplius, and (g) harpacticoid nauplius.

The images captured by the FlowCAM were semi-automatically classified into 7 target morphotypes, assisted by our existing image libraries (see examples in Fig 3). Calanoid (Calanoida), cyclopoid (Cyclopoida) and harpacticoid (Harpacticoida) nauplii were sorted from the contents of the 50 μm plankton net; whereas, calanoid (Calanoida), oithonid (Cyclopoida Oithonidae), oncaeid (Poecilostomatoida Oncaeidae), and corycaeid (Poecilostomatoida Corycaeidae) copepodites were classified from the contents of the 100 μm plankton net. Note that poecilostomatoid nauplii were not classified as an individual category due to difficulty in identifying from images. It is very likely that poecilostomatoid nauplii were classified as cyclopoid nauplii in our data given they share very similar morphologies.

**Fig 4:**
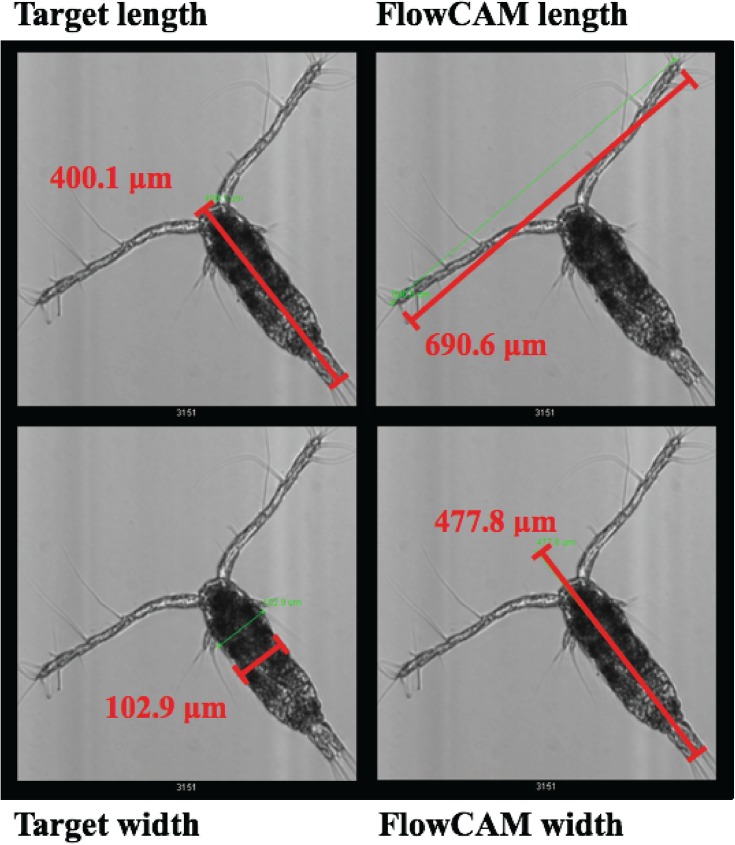
Example illustrating the inconsistency between the target length and width versus the FlowCAM image-based length and width. The target length and width of the copepod in this image are 400.1 and 102.9 μm respectively. However, the actual length and width FlowCAM measured are 690.6 and 477.8 μm respectively, which are affected by the extended copepod antenna.

Ideally, for each copepod image, both length and width can be measured by the FlowCAM image analysis software and biovolume for each individual then calculated as: biovolume = prosome length × width^2^. Unfortunately, the reported lengths and widths from the FlowCAM image analysis software do not always correspond to the minimum and maximum feret measurements claimed by the FlowCAM manual; rather, they are often affected by elongated antennae or other appendages (see example in Fig 4). Note: this problem has also been recognized by the FlowCAM manufacturer.

As a workaround, we used the Area-Based-Diameter (ABD) volume as the proxy for biovolume; this measure is only mildly affected by antennae and appendages since they occupy only a small relative area. ABD-based volume (V_ABD_) is based on the diameter of the circle obtained by arranging all the pixels deemed part of the particle into a solid circle. We then compared the V_ABD_ with biovolume calculated from manual prosome length and width measurements using the FlowCAM ruler tool on copepod images. For simplicity, we term this manual measurement of biovolume using the ruler tool as “microscopic measurement”, because this manual procedure is analogous to conventional procedures for size measurements under the microscope.

**Fig 5:**
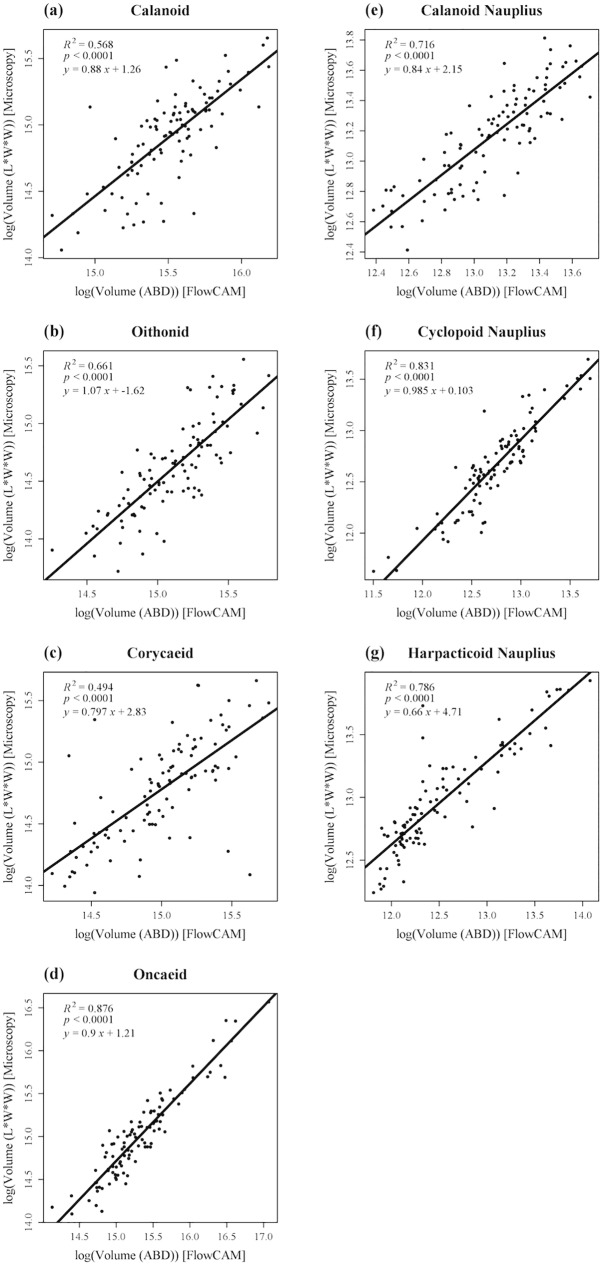
Scatter plots illustrating the relationships between the biovolumes estimated with Area-Based-Diameter (V_ABD_) from the FlowCAM versus the manual image measurements using ruler tools for the 7 dominant copepod morphotypes. A hundred individuals from each morphotype assemblage are randomly chosen. The biovolume of each individual was estimated by both V_ABD_ using the FlowCAM image analysis and “microscopic measurement” for each morphotype: (a) calanoid copepodite, (b) oithonid copepodite, (c) corycaeid copepodite, (d) oncaeid copepodite, (e) calanoid nauplius, (f) cyclopoid nauplius, and (g) harpacticoid nauplius. Linear regression analysis reveals significant correlations (p < 0.0001) between the biovolumes estimated with Area-Based-Diameter (V_ABD_) versus microscopic measurement for all morphotypes.

To test the efficacy of V_ABD_, the biovolumes of one hundred individuals of each morphotype were estimated by both “microscopic measurement” and V_ABD_ using the FlowCAM image analysis. The hundred individuals were randomly picked from pooled images of copepods sampled from the three stations. Data are provided in S1 Table. We used simple regression analysis to examine the relationship between the two measurements. Our comparison of V_ABD_ with the microscopic measurements indicates that V_ABD_ is a reasonable proxy for biovolume (Fig 5), although its efficacy is better for some taxa than others. We also provide conversion coefficients for each taxon (Table 1). This ground truthing procedure for converting V_ABD_ from FlowCAM image analysis to microscopic measurements is recommended for users targeting specific groups of organisms in specific regions. Thus, the biovolumes of zooplankton can be reasonably estimated using the regression relationships obtained from comparisons between V_ABD_ and microscopic measurements. Nevertheless, further image analysis developments may be required for improving the automatic estimation of plankton biovolume.

**Table 1:** Regression coefficients for biovolume data transformation (from FlowCAM data to Microscopy data at log scale) for 7 dominant copepod morphotypes using a linear regression model.

Copepod Morphotype	Microscopy = a × FlowCAM + b	
	a	b
Calanoid copepodite	0.88	1.26
Oithonid copepodite	1.07	-1.62
Corycaeid copepodite	0.797	2.83
Oncaeid copepodite	0.9	1.21
Calanoid nauplius	0.84	2.15
Cyclopoid nauplius	0.985	0.103
Harpacticoid nauplius	0.66	4.71

**Fig 6:**
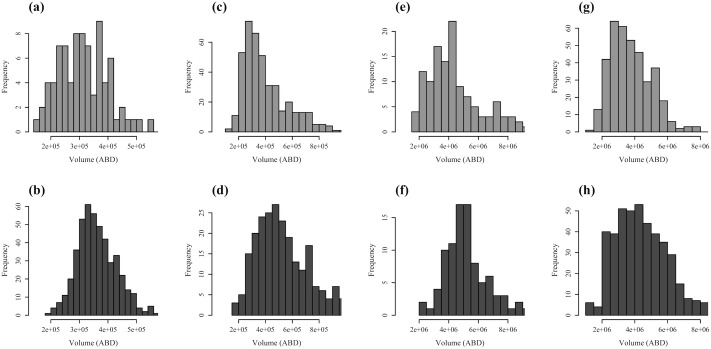
Examples of size distributions of various morphotypes based on volumes estimated from Area-Based-Diameter (V_ABD_) from the modified FlowCAM procedure before and after incubation. Size distributions were measured before and after incubation for (a, b) calanoid nauplii, (c, d) cyclopoid nauplii, (e, f) calanoid copepodites, and (g, h) oithonid copepodites. Bars denote biovolume frequency.

As an empirical demonstration of the capacity of our modified FlowCAM procedure, we provide examples of the size distribution of copepods before and after incubation in our artificial cohort experiments from station 5 (Fig 6). Comparison of these two size distributions is used to calculate growth rate (*g*) as: $g=\ln\left(\frac{W_{T}}{W_{0}}\right)/T$, where *W*_0_ is the mean carbon biomass of copepods at the beginning of incubation, *W*_*T*_ is the mean carbon biomass at the end of incubation, and *T* represents the incubation time. Here, carbon biomass can be estimated from the biovolume of plankton [19]. In this particular case of estimating growth rate, random error associated with FlowCAM image analysis is no longer a concern, because the error cancels out during the calculation of *g*. The same procedures have been applied successfully for artificial cohort experiments from other stations. As the objective of this paper is to demonstrate the procedure, we do not present those results here. Importantly, however, our procedure is reproducible, and zooplankton samples were successfully recycled without damage.

## Pros and cons of the modified FlowCAM procedure

We introduced a modified FlowCAM procedure that facilitates sample recycling and thus overcomes the limitation of small sample size in various practical situations. The advantage of automatic measurements based on the FlowCAM is that the tool is efficient and free from human errors associated with subjectivity. Based on our assessment, the modified FlowCAM procedure is 3 times more efficient than classical microscopic measurements in terms of the time-savings and manpower (Table 2). However, the disadvantage is that the size measurement is still subject to random error from automatic image analysis. In particular, when the orientation of a zooplankter is not in an upright position at the moment of image capture, the biovolume estimation is biased because the FlowCAM image analysis is based on a two-dimensional image (Table 2). We are aware that more advanced models of the FlowCAM are equipped with a syringe pump and FOV flowcell. However, these advanced setups are very expensive, contain limited syringe and flowcell sizes, and do not guarantee recycling. In contrast, our modifications are cheap and flexible, and can be used for any existing model of FlowCAM.

**Table 2:** Comparison of FlowCAM image analysis versus microscope measurements.

	FlowCAM image	Microscope measurement
Type of error	Random	Random + Personal
Target searching	Passive	Active
Sampling rate	4 hours / 1000 individuals	12 hours / 1000 individuals
Sampling randomness	High	Low
Adjusting orientation	Impossible	Possible

## Supporting information

S1 Table Biovolume data estimated with Area-Based-Diameter (V_ABD_) and the manual image measurements for the 7 dominant copepod morphotypes. (XLSX)
